# Radial-EBUS: CryoBiopsy Versus Conventional Biopsy: Time-Sample and C-Arm

**DOI:** 10.3390/ijerph19063569

**Published:** 2022-03-17

**Authors:** Paul Zarogoulidis, Christoforos S. Kosmidis, Wolfgang Hohenforst-Schmidt, Dimitrios Matthaios, Konstantinos Sapalidis, Dimitrios Petridis, Eleni-Isidora Perdikouri, Nikos Courcoutsakis, Dimitris Hatzibougias, Christos Arnaoutoglou, Lutz Freitag, Aristeidis Ioannidis, Haidong Huang, Christos Tolis, Chong Bai, J. Francis Turner

**Affiliations:** 1 Pulmonary-Oncology Department, General Clinic Euromedica, Private Hospital, 54645 Thessaloniki, Greece; 2 Surgical Department, University Hospital of Thessaloniki AHEPA, Aristotle University of Thessaloniki (AUTH), 1st St. Kiriakidi Street, 54621 Thessaloniki, Greece; dr.ckosmidis@gmail.com (C.S.K.); sapalidiskonstantinos@gmail.com (K.S.); 3 Sana Clinic Group Franken, Department of Cardiology/Pulmonology/Intensive Care/Nephrology, “Hof” Clinics, University of Erlangen, 91052 Hof, Germany; w.h-s@gmx.de; 4 Department of Medical Oncology, Rhodes General Hospital, 85133 Rhodes, Greece; dimalexpoli@yahoo.com; 5 Department of Food Science and Technology, International Hellenic University, 54621 Thessaloniki, Greece; petridis@food.teithe.gr; 6 Oncology Department, General Hospital of Volos, 38222 Volos, Greece; eigrouse@yahoo.gr; 7 Department of Radiology and Medical Imaging, University Hospital of Alexandroupolis, Democritus University of Thrace, 68100 Alexandroupolis, Greece; ncourcou@med.duth.gr; 8 Private Pathology Laboratory, “Microdiagnostics”, 54621 Thessaloniki, Greece; dhbugias@gmail.com; 9 Department of Obstetrics & Gynecology, Papageorgiou Hospital, Aristotle University of Thessaloniki, 54621 Thessaloniki, Greece; arnaoutoglou7@gmail.com; 10 Pulmonary Department, University Hospital of Zurich, 8004 Zurich, Switzerland; freitag-hemer@t-online.de; 11 Department of Respiratory & Critical Care Medicine, Changhai Hospital, The Second Military Medical University, Shanghai 200001, China; ariioann@yahoo.gr (A.I.); hhdongbs@126.com (H.H.); bai_chong@163.com (C.B.); 12 Oncoderm Private Oncology Clinic, 45221 Ioannina, Greece; oncodermcenter@gmail.com; 13 Department of Medicine, University of Tennessee Graduate School of Medicine, Knoxville, TN 37001, USA; jfrancisturner@hotmail.com

**Keywords:** radial-ebus, bronchoscopy, forceps, brush, cell-blocks, lung cancer, cryobiopsy

## Abstract

Introduction: Diagnosis of lung nodules is still under investigation. We use computed tomography scans and positron emission tomography in order to identify their origin. Patients and Methods: In our retrospective study, we included 248 patients with a single lung nodule or multiple lung nodules of size ≥1 cm. We used a radial-endobronchial ultrasound and a C-Arm. We used a 1.1 mm cryoprobe versus a 22G needle vs. forceps/brush. We compared the sample size of each biopsy method with the number of cell-block slices. Results: Central lesions indifferent to the method provided the same mean number of cell-block slices (0.04933–0.02410). Cryobiopsies provide less sample size for peripheral lesions due to the higher incidence of pneumothorax (0.04700–0.02296). Conclusion: The larger the lesion ≥2 cm, and central, more cell-blocks are produced indifferent to the biopsy method (0.13386–0.02939). The time of the procedure was observed to be less when the C-Arm was used as an additional navigation tool (0.14854–0.00089).

## 1. Introduction

In the past 15 years, technology has provided us with novel diagnostic equipment and techniques for lung cancer. Lung cancer is usually diagnosed at a late stage due to a lack of early disease symptoms. We still do not have early blood biomarkers, such as in the case of prostate cancer, gastrointestinal cancer, and breast cancer [1,2,3]. Therefore, we have focused our efforts towards prevention with computed tomography scans of patients ≥50 years of age, smokers, former smokers, and patients with a known cancer family medical history [4,5]. Patients with non-small cell lung (NSCLC) with inoperable disease status need an investigation of their cancer genome with next-generation sequencing (NGS) [6,7]. The reason for this is to provide the appropriate treatment by investigating whether the tumor expresses the epidermal growth factor receptor (EGFR), anaplastic lymphoma kinase (ALK), proto-oncogene 1 (ROS-1), proto-oncogene B-Raf (BRAF), and programmed death-ligand 1 (PD-L1) [6,8,9,10,11,12]. Based on the findings, we administer either tyrosine kinase inhibitor (TKIS), immunotherapy, chemotherapy, or a combination of those drugs [6,8]. Tissue biopsies or cell-block biopsies provide us with efficient material in order to investigate all these genes and protein expressions [11,13]. Next-generation sequencing (NGS) is an efficient method that can identify simultaneously several genes, although it might take some days [6,14]. Currently, the IHC 22C3pharmDx DAKO (Companion diagnostic system, Dako, Denmark, EU) has been validated and is used in everyday clinical practice for the evaluation of PD-L1 expression, and in many countries, the administration of immunotherapy is impossible if the expression has been determined with another clone [15]. Regarding small cell lung cancer (SCLC), we do not need for now an investigation of different gene expressions, although there are several ongoing studies in the field. Therefore, we need a large sample size in order to perform next-generation sequencing and avoid obtaining necrotic tissue, as could happen with squamous cell carcinoma.

**Figure 1:**
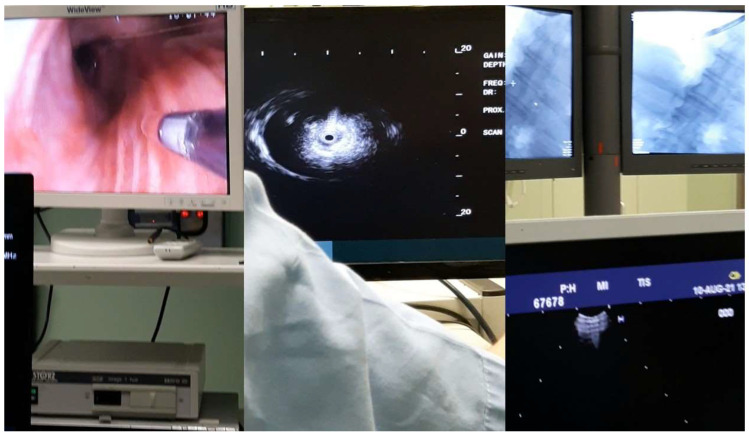
**Left** and **middle**: the Fuji radial probe; **right**: image from the C-Arm up and down image from the convex probe (PENTAX).

**Figure 2:**
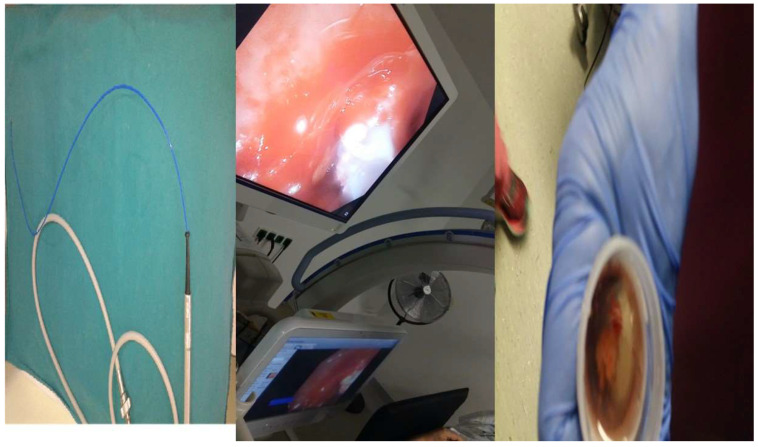
**Left**: ERBE 1.1 mm cryoprobe; **middle**: image during the biopsy; and **right**: sample specimen.

**Figure 3:**
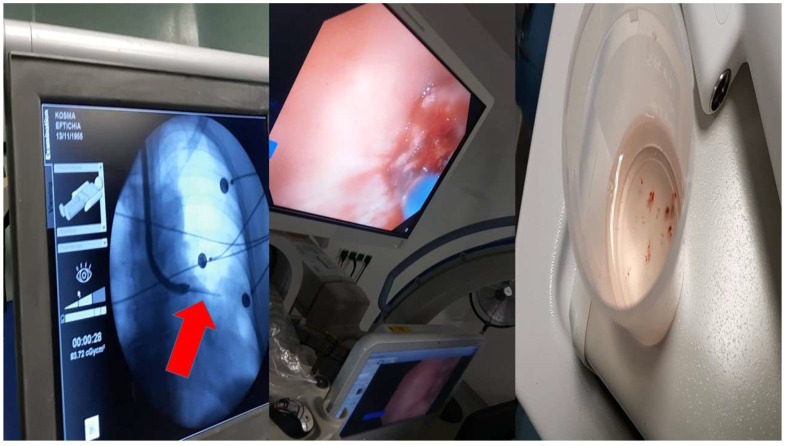
**Left**: C-Arm with the forceps outside the working channel of the bronchoscope during biopsy; **middle**: image during biopsy; and **right**: sample specimen.

**Figure 4:**
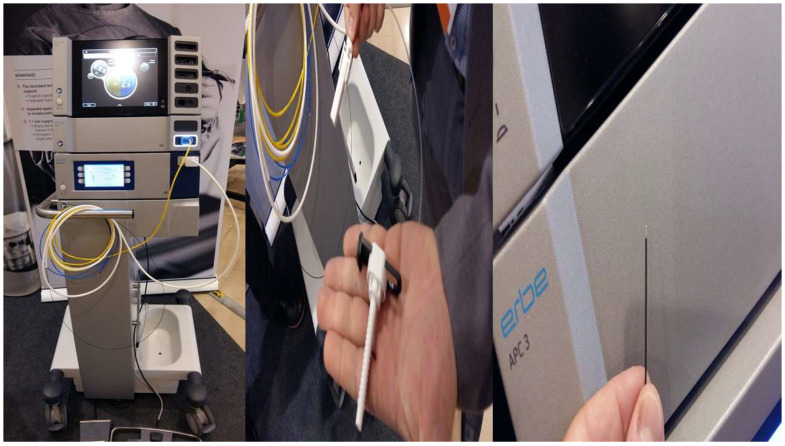
**Left**: ERBE 2 platform; **middle** and **right**: the probe 1.1 mm.

Currently, we are using a computed tomography (CT) scan for biopsy, radial-endobronchial ultrasound, convex-endobronchial ultrasound, electromagnetic navigation systems (superDimension™, Medtronic, Archemedes^®^, Bronchus), and transthoracic convex probe guided biopsies [13,16,17]. Additionally, we have the rapid on-site evaluation (ROSE) technique, used as a fast on-site form of diagnosis, and the CellVizio system, which is also a rapid on-site diagnostic equipment [18,19]. Elastography is a method for the rapid on-site evaluation of a lesion in order to investigate benignancy or malignancy. Moreover, we can evaluate the best site to puncture and acquire samples, although targeting a specific area is very difficult in lesions ≤3 cm [20,21]. However, after several studies, again it was observed that biopsy is necessary to provide a safe result. Elastography can be used only to identify the stiffness of a lesion—it is a useful tool to avoid necrosis. All techniques have their advantages and disadvantages when it comes to the sample size. The method of sedation plays a crucial role in efficiently performing a biopsy. When the patient is under optimal circumstances, a biopsy can be performed more rapidly and with fewer complications. Moreso, even we have complications, they can be dealt with quickly. The type of sedation differs from center to center and from patient to patient based on how many lesions or lymph node stations we have to biopsy. We need at least four biopsies from every lesion with a 22G needle in order to acquire sufficient samples for diagnosis and next-generation sequencing (NGS). Sedation is one of the main reasons why there are efficiency differences in interventional pulmonary centers. In centers without an anesthesiologist present, the sedation is mild, and therefore, the procedure is longer and with less sample size obtained by the users in order to avoid possible complications. However, for early disease with just a single nodule, large sample sizes are not necessary, because we need just enough material for diagnosis and not next-generation sequencing. In the case of a single nodule or nodules ≤2 cm, a biopsy is essential since the positron emission tomography (PET-CT) can be falsely negative because of the low metabolic rate of the underlying disease. Regarding peripheral nodules, we use the radial-ebus with the additional navigation of a C-Arm, or we use electromagnetic navigation. We usually use a brush or forceps in order to acquire a sample, however, cryobiopsy with small 1.1 mm cryoprobes have provided us an additional and useful tool for diagnosis [22,23,24,25,26,27,28,29,30]. In several previous publications, we have evaluated the sample size of different needles and needle types by using cell-blocks and the number of cell-block slices [13,31]. In the current clinical study, we evaluated the sample size of cryobiopsies versus forceps + brush. Moreover, we recorded the synergistic effect of the C-Arm with the radial-ebus as a guidance tool along with the location of the nodules (peripheral vs. central location) and the time of each procedure. We investigated in depth how the following factors interact: (a) time of the procedure, (b) method of biopsy (forceps, cryoprobe, (c) C-Arm guidance system, (d) location of the lesion. We provided an algorithm that indicates, based on the location of the tumor, the best methodology in order to acquire a large sample size (Figure 1, Figure 2, Figure 3 and Figure 4).

## 2. Patients and Methods

In total, 248 patients were enrolled in our study. All patients had either one or several pulmonary nodules diagnosed with computed tomography scan, and then positron emission tomography (PET-CT) was performed. All patients in our study were included based on the fact that they had nodules ≥1 cm. Therefore, we performed biopsies only on lesions that were positive with a PET-CT or negative; however, we performed a biopsy since there was a high suspicion of malignancy. Our study was retrospective, and all patients were ≥18 years of age. Our study was approved by the investigational review board (IRB) of our institution (Aristotle University of Thessaloniki, Greece). We used a Pentax EB-1570 endoscope with an EPK-1000 and a Fuji radial-endobronchial ultrasound probe. All lesions varied from 1 cm to 4 cm and the time of each procedure was recorded. Moreover, we recorded whether a C-Arm was used as an additional tool for fast guidance and diagnosis. Forceps, brush, 22G needles, and a cryoprobe 1.1 mm from ERBE were used as a tool for biopsy (Figure 1, Figure 2, Figure 3 and Figure 4). The length of each procedure was recorded, since all our procedures were conducted in a specially designed surgical suite for upgraded endoscopic procedures. The time length was recorded and divided into three periods: ≤20 min, 20–40 min, and ≥40 min. All patients had anesthesia and were intubated with a tracheal tube n.8 to 9, or a rigid bronchoscope Storz (12) with an inner working channel of 11 mm, or we used a fast-track laryngeal mask. For all our patients, we used jet ventilation.

All patients were eligible for anesthesia.

## 3. Statistical Analysis

Tumor location and lesion sizes were the parameters of main interest. Secondarily C-Arm and the time interval of each operation on the one hand, and the method of lung penetration and the number of slices, on the other hand, thus formed two different approaches for statistical purposes. In both groups, a multiple correspondence analysis among variable categories was applied, while the number of slices was entered as a supplementary variable in the analysis. The particular contribution of each of the combined categories to the formation of the first two dimensions was thoroughly examined. Multiple correspondence analysis (MCA) takes multiple categorical variables and seeks to identify associations between the levels of those variables (LeRoux and Rouanet (2010) [32]. MCA extends correspondence analysis from two variables to many. It can be thought of as analogous to principal component analysis (PCA) for quantitative variables. Similar to other multivariate methods, it is a dimension-reducing method; it represents the data as points in 2- or 3-dimensional space, plotting the first two major dimensions, known as inertia, and reflecting the relative variation accounted for in the dimensions. The contribution of each category (arrayed in rows or columns) to the inertia of a dimension is calculated as follows:

contribution = (mass)(coordinate)2/(dimension inertia)

and can be thought of as analogous to the correlation between the variables and the first two components in PCA.

All the variables involved in the study were categorized according to the first column of Table 1. All patients were included in the statistical analysis.

## 4. Results

**Figure 5:**
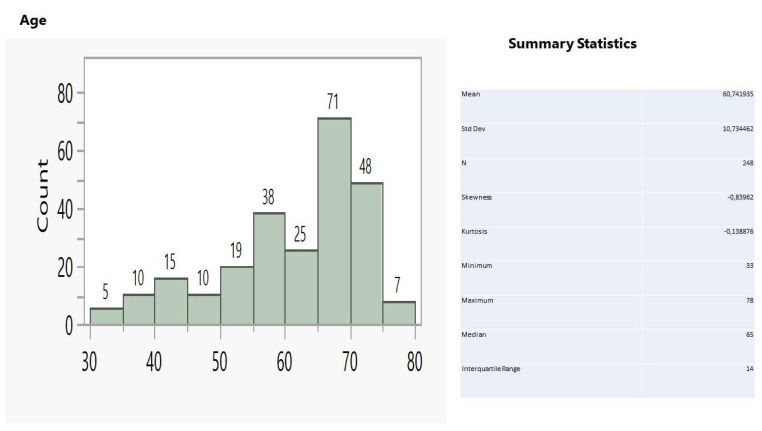
Age distribution and relevant statistics.

In the sample of 248 patients suffering from lung cancer, ages between 55 and 75 years old dominated in the age distribution (Figure 5), constituting 73.4%, while males prevailed over females at a ratio of 1.6:1 (153/95).

The most frequent categories encountered in the study (Table 1) were the lesion size 1–2 cm (38.7%), peripheral tumor location (51.2%), less than 20 min operative time (51.2%), no C-Arm operation (52.8%), and lung tissue penetration with forceps (47.6%). For the cross-tabulated variables belonging to the first group, the combination of a central tumor location, with a 2–3 cm lesion size, zero C-Arm, and a 20 min operation were recorded in 49 cases (17.2%), and that with a peripheral location, with a 1–2 cm lesion size, the presence of the C-Arm and a 41–60 min operation was recorded in 38 cases (15.3%). From the table of the second group of interest, 34 cases (13.7%) were both counted in two combined categories: first with a central tumor location, with a 2–3 cm lesion size, forceps use, and 15 slices; the second was with a peripheral location, 0–1 cm, cryoprobe use, and 8 slices. It is noteworthy to mention that the mean slices were higher in all combined categories connected with the central tumor location.

**Figure 6:**
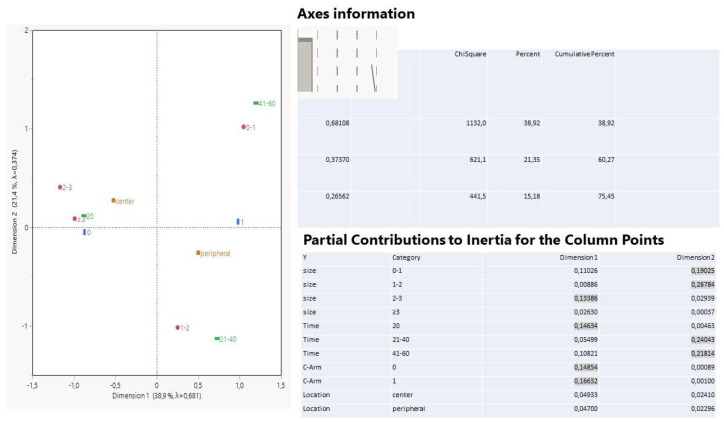
Multiple correspondence analysis among categories of four variables: tumor location, lesion size, operation of C-Arm, and time duration. Points positioned in close approximation indicate strong affiliation and distant points, from the center, indicate a strong effect on either dimension. The highlighted coordinates reveal a comparatively higher correlation with each dimension.

Concerning the first approach of interest, the multiple correspondence analysis revealed remarkable results (Figure 6).

The first two dimensions in the correspondence biplot explain 60.3% of the total variation (inertia) in which the lesion size of 2–3 cm, with a 30 min operative time, and the C-Arm present or absent, were the most important contributors and were responsible for the formation of dimension 1 (partial contribution 60%). For the formation of the second dimension, the lesion sizes 0–1 and 1–2 cm, and an operative time of 21–40 and 41–60 min, were the best contributors, totaling 92%. In the correspondence plot, points (categories) close to the axes and distant apart, contribute the most, while points arranged nearby form clusters of similar attitudes. Thus, on the left half of the graph, a cluster is formed with common characteristics of higher levels of lesion size (≥2 cm) encountered in central tumors that needed a short operative time (max 20 min) and no C-Arm vision. On the right part of the plot, three clusters signal that the peripheral tumors are strongly connected with C-Arm examination; the smallest lesion size (0–1 cm) is inevitably related to a long operative time (41–60 min) and goes without saying, while the middle levels of lesion size (1–2) and an operative time of 21–40 min are jointly connected.

**Figure 7:**
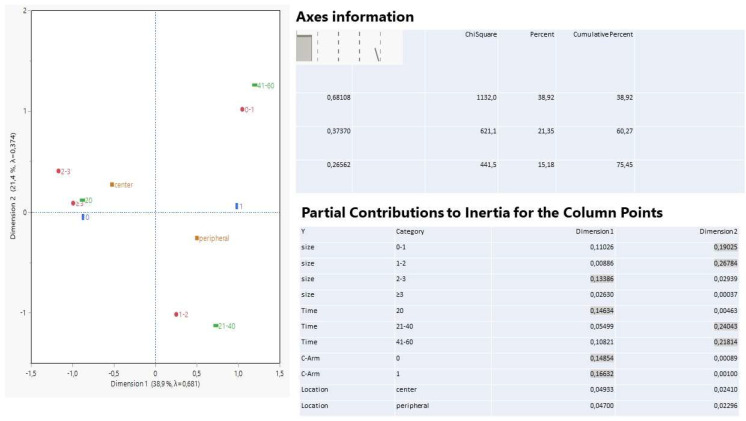
Multiple correspondence analysis among categories of four variables: tumor location, lesion size, method of lung penetration, and the number of slices entered as a supplementary variable. Points positioned in close approximation indicate strong affiliation, and distant points from the center indicate a strong effect on either dimension. The highlighted coordinates reveal a comparatively higher correlation with each dimension.

In the next group of interest, the different techniques of lung tissue penetration and the number of slices created form particular patterns in the biplot of multiple correspondence analysis (Figure 7).

Both dimensions explain 48.6% of the total inertia, and the categories of cryoprobe insertion and lesion size 0–1 and 2–3 cm contribute 58% to the formation of the first dimension, while penetration via a 22G needle and forceps, together with the largest lesion size, (≥3 cm) contribute 83% to the formation of dimension 2. There appears a shift of gradually increasing numbers of slices from the left to the right direction along the first dimension of the correspondence biplot, highlighting particular positions of category points with proximate values of slices. Specifically, the cryoprobe operation, together with the smallest lesion size (0–1 cm) is commonly encountered and associated with a small number of slices. The latter increases up to 8–9 slices in the middle region between peripheral tumors and brush insertion, reaching 15 slices for lesions that are 1–2 cm, 16–18 slices when forceps are used, and 19–20 slices in the lesion size of 2–3 cm. The central tumors are surrounded by a high number of slices, and interestingly, the highest lesion size (≥3 cm) is affiliated with only 4 and 5 slices, showing also a loose connection with the 22G needle insertion, which also approaches high levels of slices.

## 5. Discussion

Single nodules are considered a major diagnostic issue in everyday clinical practice. We use the PET-CT as an initial diagnostic tool for their evaluation [33]. The positive results provide us with the solution, however, the major issue is false-negative results. The limitations of the PET-CT technique are well known where a malignancy with a low metabolic rate of ki-67 ≤10 usually has low FDG uptake and is usually growing at a very low rate through a 10-year period of time. These malignancies are usually adenocarcinomas or neuroendocrine tumors [34,35]. Therefore, we need efficient diagnostic tools, which we have. We investigated in depth how the following factors interact: (a) time of the procedure, (b) method of biopsy (forceps, cryoprobe), (c) C-Arm guidance system, (d) location of the lesion. We provided an algorithm that indicates, based on the location of the tumor, the best methodology in order to acquire a large sample size.

A single nodule can be punctured under CT guidance with different needles from 18G to 22G. Larger bore needles, such as the 16G needle increase the incidence of pneumothorax. In any case, biopsy under CT guidance has a higher incidence of pneumothorax, even in the most experienced centers [36]. Therefore, we prefer the endoscopic approach if possible. The electromagnetic navigation systems have the disadvantage of an increased price of the main equipment and probes, which are not reusable. Nowadays, apart from the superDimension™, Medtronic, Archemedes^®^, and Bronchus the Monarch^®^, a platform for AURIS Health is also available. Therefore, the radial-endobronchial ultrasound with a C-Arm is cheaper, along with the rapid on-site evaluation (ROSE), which is also a cheap technique, and the diagnosis of a single nodule or nodules is more feasible. The confocal microscope is another rapid on-site evaluation; however, with a very high cost when compared to the ROSE technique performed by a cytologist or pulmonary physician. In any case, every doctor will use their equipment in the best possible way in order to accomplish the diagnosis. In our study, we compared the biopsy sample from cryobiopsies with the forceps/brush sample and 22G needle sample. We concluded that the time of a procedure depends on the location of the lesion, size of the lesion, and use of the C-Arm. The anesthesia provided to each patient was the same, thus the condition for each patient was the same for the medic to perform under optimal circumstances. The larger the lesion ≥2–4 cm, central lesions compared to peripheral, and the use of the C-Arm provided more sample material (cell-block slices). When we compared the cryobiopsies to forceps/brush and the 22G needle, we concluded that the number of slices produced was less for peripheral lesions, possibly due to the fact that blind biopsies with a cryoprobe in the lung periphery can cause pneumothorax, and therefore, we reproduced the cryobiopsy procedure fewer times. The number of slices was higher with the forceps/brush compared to 22G needles, again due to the limitation of the size and location—the number of punctures was less with the 22G needles. It is known that for central lesions, cryobiopsies can cause larger hemoptysis than forceps and 22G needles due to the larger vessel diameter in the central region of the lung. On the other hand, cryobiopsies increase the incidence of pneumothorax in the case of peripheral lesions. Moreover, after discussion with our pathology department, we concluded that the cryobiopsies have an issue since the −80 °C partially destroy the tissue sample. Hemoptysis events were higher, and were observed to be higher for the cryobiopsies and were treated with a hemostatic polymer powder sprayed with a special pump. No pneumothorax was observed. A major limitation of our study was the lack of rapid on-site evaluation (ROSE), which we did not use due to a lack of funding. We propose that the tools used for biopsy are up to the experience of the center and that, if available, electromagnetic navigation tools can certainly assist in lowering the time of the diagnostic procedure since they are a more complete guidance system.

## Figures and Tables

**Table 1:** Individual and cross-tabulation of the six parameters of interest: tumor size (cm) and location, operative time (min), use of C-Arm, method of penetration, and the number of slices. Tumor location and lesion size are presented twice in the tables.

**Size 2**	**N**	**Location 2**	**Size 2**	**C-Arm**	**Time 2**	**N**				**N Slices**	
0–1	68	Center	0–1	1	20	5	**Location 2**	**Size 2**	**Method 2**	**Mean**	**N**
1–2	96				21–40	8	Center	0–1	22GNeedle	11	3
2–3	66				41–60	15			Forceps	13	7
≥3	18		1–2	0	20	17			CryoProbe	10	18
**Location 2**					21–40	13		1–2	22GNeedle	13	9
Center	121			1	20	5			Forceps	13	27
Peripheral	127				41–60	1		2–3	22GNeedle	14	15
**Time 2**			2–3	0	20	49			Forceps	15	34
20	127		≥3	0	20	8		≥3	22GNeedle	12	8
21–40	70		0–1	0	20	3		0–1	Forceps	7	4
41–60	51			1	21–40	10			Brush	2	2
**C-Arm**					41–60	27			CryoProbe	8	34
0	131		1–2	0	20	13		1–2	22GNeedle	7.5	4
1	117				21–40	1			Forceps	8.4	29
**Method 2**				1	21–40	38			Brush	8.5	11
22GNeedle	49				41–60	8			CryoProbe	8.6	16
Forceps	118		2–3	0	20	17		2–3	Forceps	9	17
Brush	13		≥3	0	20	10		≥3	22GNeedle	4.3	10
Cryoprobe	68										

## Data Availability

Data for this study can be provided upon request from the corresponding author.
